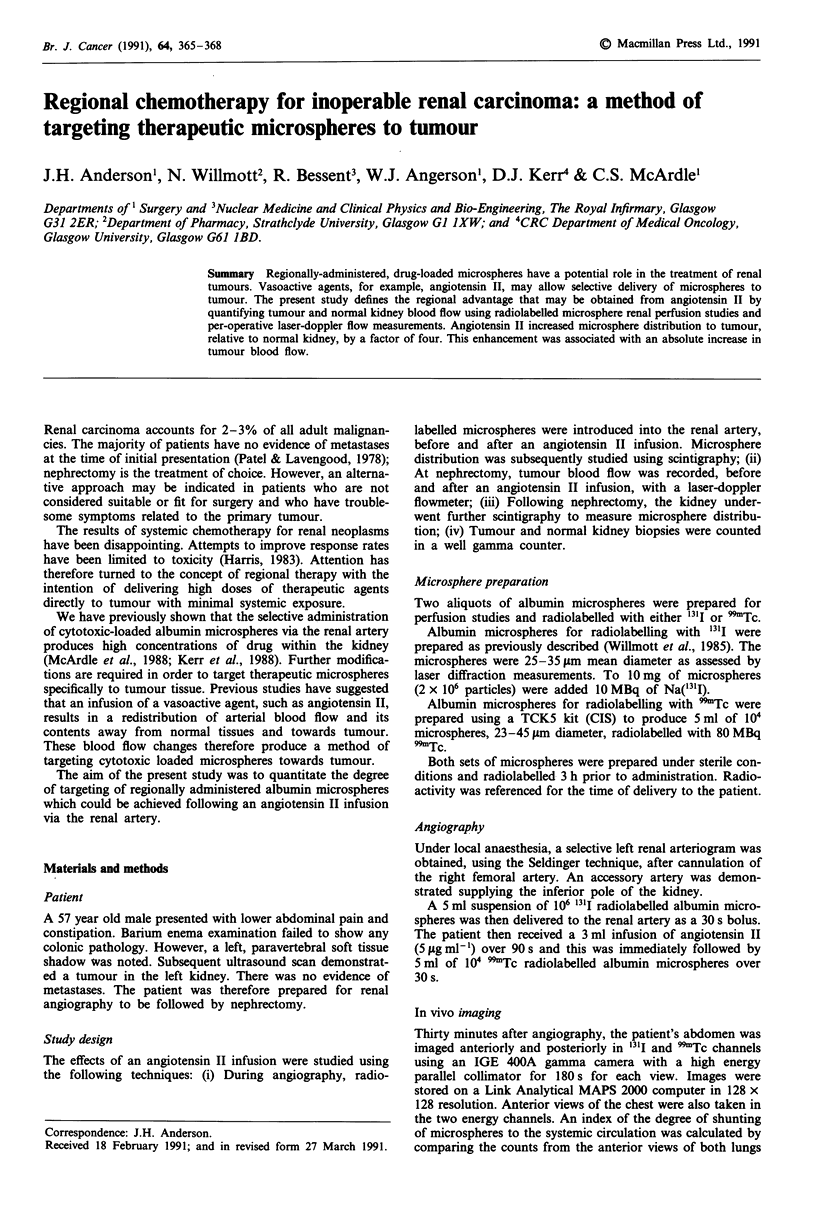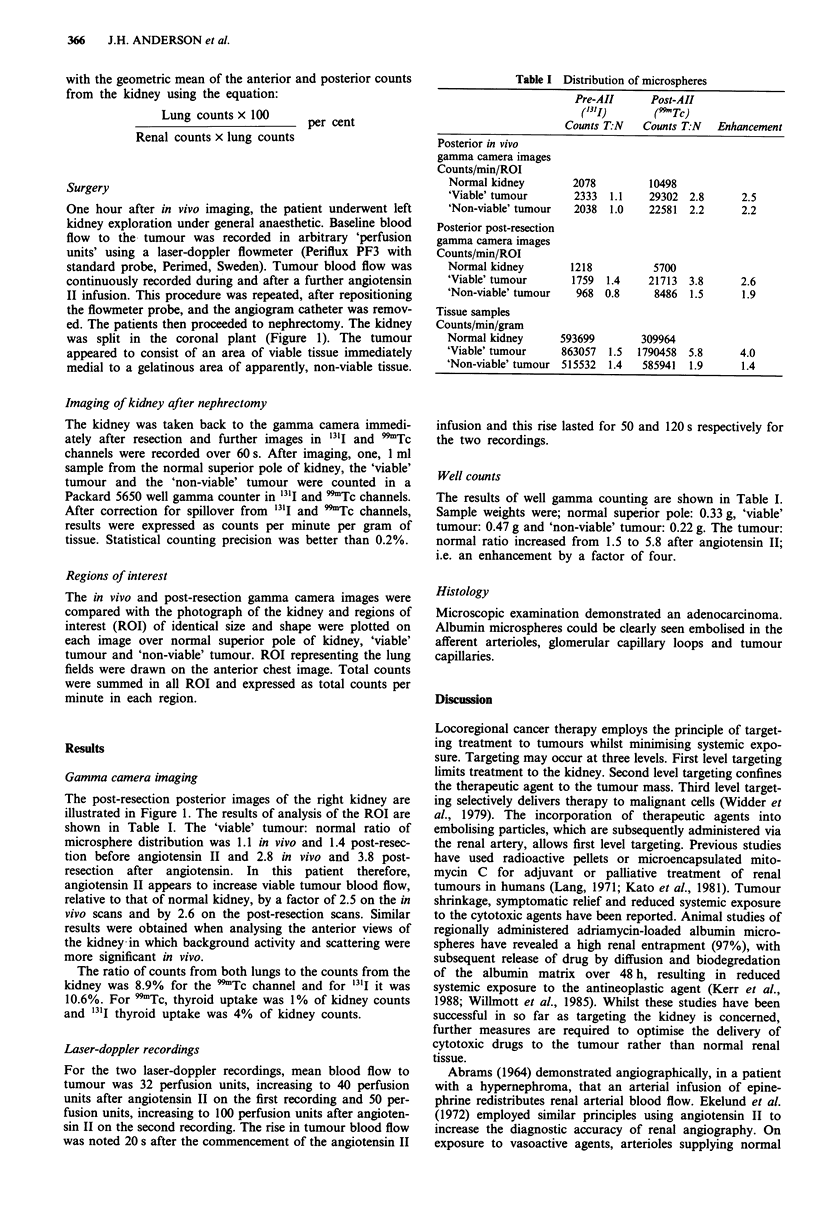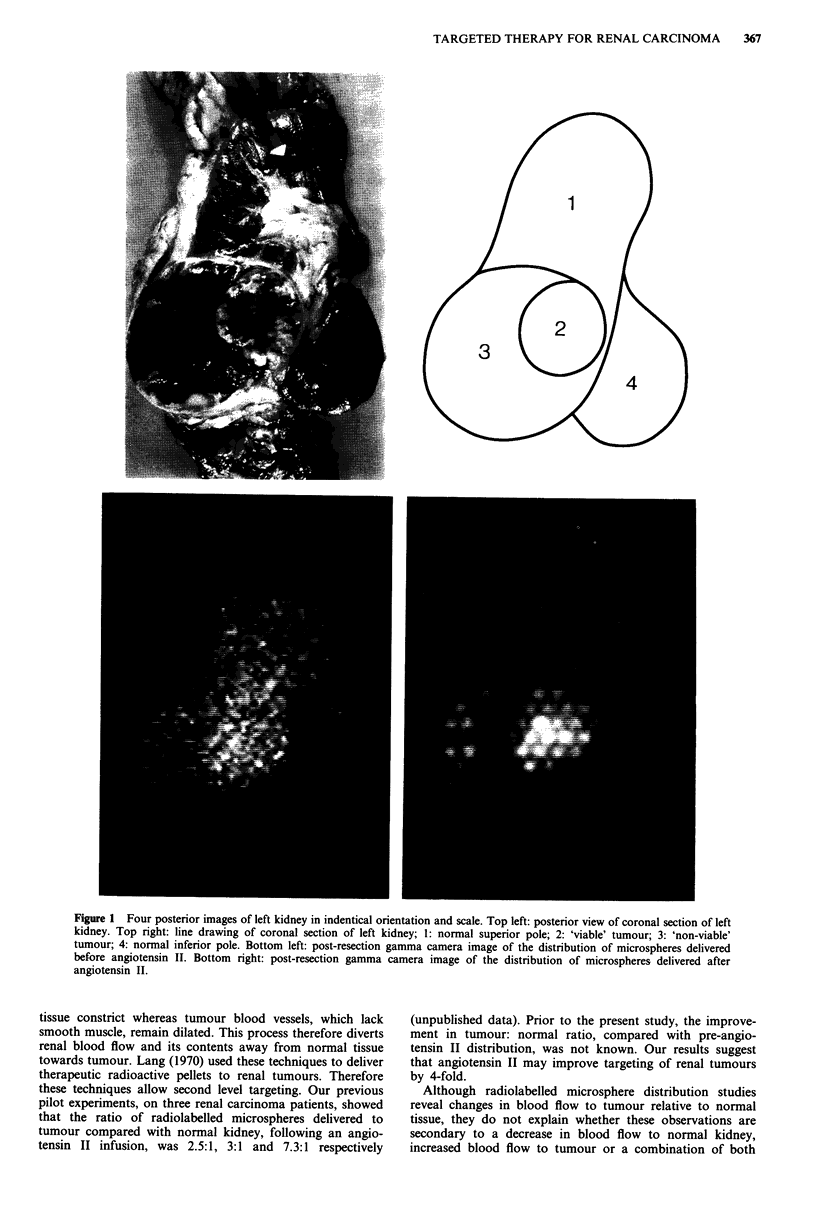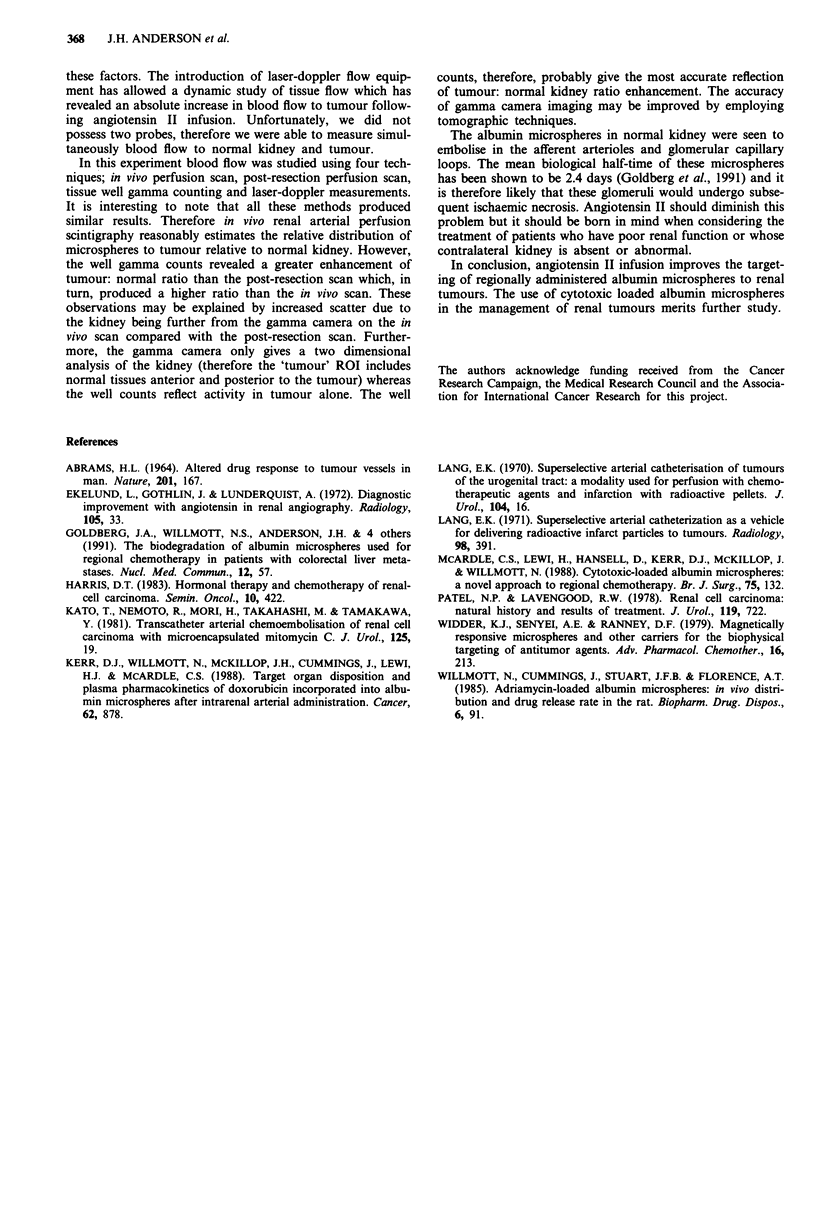# Regional chemotherapy for inoperable renal carcinoma: a method of targeting therapeutic microspheres to tumour.

**DOI:** 10.1038/bjc.1991.308

**Published:** 1991-08

**Authors:** J. H. Anderson, N. Willmott, R. Bessent, W. J. Angerson, D. J. Kerr, C. S. McArdle

**Affiliations:** Department of Surgery, Royal Infirmary, Glasgow.

## Abstract

**Images:**


Br. J. Cancer (1991), 64, 365-368 © Macmillan Press Ltd., 1991

### Regional chemotherapy for inoperable renal carcinoma: a method of targeting therapeutic microspheres to tumour

J.H. Anderson[1], N. Willmott[2], R. Bessent[3], W.J. Angerson[1], D.J. Kerr[4] & C.S. McArdle[1]

*Departments of [1] Surgery and [3]Nuclear Medicine and Clinical Physics and Bio-Engineering, The Royal Infirmary, Glasgow G31 2ER; [2]Department of Pharmacy, Strathclyde University, Glasgow G1 1XW; and [4]CRC Department of Medical Oncology, Glasgow University, Glasgow G61 1BD.*

**Summary** Regionally-administered, drug-loaded microspheres have a potential role in the treatment of renal tumours. Vasoactive agents, for example, angiotensin II, may allow selective delivery of microspheres to tumour. The present study defines the regional advantage that may be obtained from angiotensin II by quantifying tumour and normal kidney blood flow using radiolabelled microsphere renal perfusion studies and per-operative laser-doppler flow measurements. Angiotensin II increased microsphere distribution to tumour, relative to normal kidney, by a factor of four. This enhancement was associated with an absolute increase in tumour blood flow.

Renal carcinoma accounts for 2-3% of all adult malignancies. The majority of patients have no evidence of metastases at the time of initial presentation (Patel & Lavengood, 1978); nephrectomy is the treatment of choice. However, an alternative approach may be indicated in patients who are not considered suitable or fit for surgery and who have troublesome symptoms related to the primary tumour.

The results of systemic chemotherapy for renal neoplasms have been disappointing. Attempts to improve response rates have been limited to toxicity (Harris, 1983). Attention has therefore turned to the concept of regional therapy with the intention of delivering high doses of therapeutic agents directly to tumour with minimal systemic exposure.

We have previously shown that the selective administration of cytotoxic-loaded albumin microspheres via the renal artery produces high concentrations of drug within the kidney (McArdle *et al.*, 1988; Kerr *et al.*, 1988). Further modifications are required in order to target therapeutic microspheres specifically to tumour tissue. Previous studies have suggested that an infusion of a vasoactive agent, such as angiotensin II, results in a redistribution of arterial blood flow and its contents away from normal tissues and towards tumour. These blood flow changes therefore produce a method of targeting cytotoxic loaded microspheres towards tumour.

The aim of the present study was to quantitate the degree of targeting of regionally administered albumin microspheres which could be achieved following an angiotensin II infusion via the renal artery.

### Materials and methods

### *Patient*

A 57 year old male presented with lower abdominal pain and constipation. Barium enema examination failed to show any colonic pathology. However, a left, paravertebral soft tissue shadow was noted. Subsequent ultrasound scan demonstrated a tumour in the left kidney. There was no evidence of metastases. The patient was therefore prepared for renal angiography to be followed by nephrectomy.

### *Study design*

The effects of an angiotensin II infusion were studied using the following techniques: (i) During angiography, radiolabelled microspheres were introduced into the renal artery, before and after an angiotensin II infusion. Microsphere distribution was subsequently studied using scintigraphy; (ii) At nephrectomy, tumour blood flow was recorded, before and after an angiotensin II infusion, with a laser-doppler flowmeter; (iii) Following nephrectomy, the kidney underwent further scintigraphy to measure microsphere distribution; (iv) Tumour and normal kidney biopsies were counted in a well gamma counter.

### *Microsphere preparation*

Two aliquots of albumin microspheres were prepared for perfusion studies and radiolabelled with either $^{131}I$ or $^{99m}Tc$.

Albumin microspheres for radiolabelling with $^{131}I$ were prepared as previously described (Willmott *et al.*, 1985). The microspheres were 25-35 $\mu m$ mean diameter as assessed by laser diffraction measurements. To 10 mg of microspheres ($2 \times 10^6$ particles) were added 10 MBq of $Na(^{131}I)$.

Albumin microspheres for radiolabelling with $^{99m}Tc$ were prepared using a TCK5 kit (CIS) to produce 5 ml of $10^4$ microspheres, 23-45 $\mu m$ diameter, radiolabelled with 80 MBq $^{99m}Tc$.

Both sets of microspheres were prepared under sterile conditions and radiolabelled 3 h prior to administration. Radioactivity was referenced for the time of delivery to the patient.

### *Angiography*

Under local anaesthesia, a selective left renal arteriogram was obtained, using the Seldinger technique, after cannulation of the right femoral artery. An accessory artery was demonstrated supplying the inferior pole of the kidney.

A 5 ml suspension of $10^6$ $^{131}I$ radiolabelled albumin microspheres was then delivered to the renal artery as a 30 s bolus. The patient then received a 3 ml infusion of angiotensin II ($5 \mu g\, ml^{-1}$) over 90 s and this was immediately followed by 5 ml of $10^4$ $^{99m}Tc$ radiolabelled albumin microspheres over 30 s.

### In vivo *imaging*

Thirty minutes after angiography, the patient's abdomen was imaged anteriorly and posteriorly in $^{131}I$ and $^{99m}Tc$ channels using an IGE 400A gamma camera with a high energy parallel collimator for 180 s for each view. Images were stored on a Link Analytical MAPS 2000 computer in 128 × 128 resolution. Anterior views of the chest were also taken in the two energy channels. An index of the degree of shunting of microspheres to the systemic circulation was calculated by comparing the counts from the anterior views of both lungs

Correspondence: J.H. Anderson.
Received 18 February 1991; and in revised form 27 March 1991.

with the geometric mean of the anterior and posterior counts from the kidney using the equation:

$$\frac{\text{Lung counts} \times 100}{\text{Renal counts} \times \text{lung counts}} \text{ per cent}$$

*Surgery*

One hour after *in vivo* imaging, the patient underwent left kidney exploration under general anaesthetic. Baseline blood flow to the tumour was recorded in arbitrary 'perfusion units' using a laser-doppler flowmeter (Periflux PF3 with standard probe, Perimed, Sweden). Tumour blood flow was continuously recorded during and after a further angiotensin II infusion. This procedure was repeated, after repositioning the flowmeter probe, and the angiogram catheter was removed. The patients then proceeded to nephrectomy. The kidney was split in the coronal plant (Figure 1). The tumour appeared to consist of an area of viable tissue immediately medial to a gelatinous area of apparently, non-viable tissue.

*Imaging of kidney after nephrectomy*

The kidney was taken back to the gamma camera immediately after resection and further images in $^{131}$I and $^{99m}$Tc channels were recorded over 60 s. After imaging, one, 1 ml sample from the normal superior pole of kidney, the 'viable' tumour and the 'non-viable' tumour were counted in a Packard 5650 well gamma counter in $^{131}$I and $^{99m}$Tc channels. After correction for spillover from $^{131}$I and $^{99m}$Tc channels, results were expressed as counts per minute per gram of tissue. Statistical counting precision was better than 0.2%.

*Regions of interest*

The *in vivo* and post-resection gamma camera images were compared with the photograph of the kidney and regions of interest (ROI) of identical size and shape were plotted on each image over normal superior pole of kidney, 'viable' tumour and 'non-viable' tumour. ROI representing the lung fields were drawn on the anterior chest image. Total counts were summed in all ROI and expressed as total counts per minute in each region.

### Results

*Gamma camera imaging*

The post-resection posterior images of the right kidney are illustrated in Figure 1. The results of analysis of the ROI are shown in Table I. The 'viable' tumour: normal ratio of microsphere distribution was 1.1 *in vivo* and 1.4 post-resection before angiotensin II and 2.8 *in vivo* and 3.8 post-resection after angiotensin. In this patient therefore, angiotensin II appears to increase viable tumour blood flow, relative to that of normal kidney, by a factor of 2.5 on the *in vivo* scans and by 2.6 on the post-resection scans. Similar results were obtained when analysing the anterior views of the kidney in which background activity and scattering were more significant *in vivo*.

The ratio of counts from both lungs to the counts from the kidney was 8.9% for the $^{99m}$Tc channel and for $^{131}$I it was 10.6%. For $^{99m}$Tc, thyroid uptake was 1% of kidney counts and $^{131}$I thyroid uptake was 4% of kidney counts.

*Laser-doppler recordings*

For the two laser-doppler recordings, mean blood flow to tumour was 32 perfusion units, increasing to 40 perfusion units after angiotensin II on the first recording and 50 perfusion units, increasing to 100 perfusion units after angiotensin II on the second recording. The rise in tumour blood flow was noted 20 s after the commencement of the angiotensin II infusion and this rise lasted for 50 and 120 s respectively for the two recordings.

**Table I** Distribution of microspheres

| | Pre-AII ($^{131}I$) Counts | T:N | Post-AII ($^{99m}Tc$) Counts | T:N | Enhancement |
|---|---|---|---|---|---|
| Posterior *in vivo* gamma camera images Counts/min/ROI | | | | | |
| Normal kidney | 2078 | | 10498 | | |
| 'Viable' tumour | 2333 | 1.1 | 29302 | 2.8 | 2.5 |
| 'Non-viable' tumour | 2038 | 1.0 | 22581 | 2.2 | 2.2 |
| Posterior post-resection gamma camera images Counts/min/ROI | | | | | |
| Normal kidney | 1218 | | 5700 | | |
| 'Viable' tumour | 1759 | 1.4 | 21713 | 3.8 | 2.6 |
| 'Non-viable' tumour | 968 | 0.8 | 8486 | 1.5 | 1.9 |
| Tissue samples Counts/min/gram | | | | | |
| Normal kidney | 593699 | | 309964 | | |
| 'Viable' tumour | 863057 | 1.5 | 1790458 | 5.8 | 4.0 |
| 'Non-viable' tumour | 515532 | 1.4 | 585941 | 1.9 | 1.4 |

*Well counts*

The results of well gamma counting are shown in Table I. Sample weights were; normal superior pole: 0.33 g, 'viable' tumour: 0.47 g and 'non-viable' tumour: 0.22 g. The tumour: normal ratio increased from 1.5 to 5.8 after angiotensin II; i.e. an enhancement by a factor of four.

*Histology*

Microscopic examination demonstrated an adenocarcinoma. Albumin microspheres could be clearly seen embolised in the afferent arterioles, glomerular capillary loops and tumour capillaries.

### Discussion

Locoregional cancer therapy employs the principle of targeting treatment to tumours whilst minimising systemic exposure. Targeting may occur at three levels. First level targeting limits treatment to the kidney. Second level targeting confines the therapeutic agent to the tumour mass. Third level targeting selectively delivers therapy to malignant cells (Widder *et al.*, 1979). The incorporation of therapeutic agents into embolising particles, which are subsequently administered via the renal artery, allows first level targeting. Previous studies have used radioactive pellets or microencapsulated mitomycin C for adjuvant or palliative treatment of renal tumours in humans (Lang, 1971; Kato *et al.*, 1981). Tumour shrinkage, symptomatic relief and reduced systemic exposure to the cytotoxic agents have been reported. Animal studies of regionally administered adriamycin-loaded albumin microspheres have revealed a high renal entrapment (97%), with subsequent release of drug by diffusion and biodegredation of the albumin matrix over 48 h, resulting in reduced systemic exposure to the antineoplastic agent (Kerr *et al.*, 1988; Willmott *et al.*, 1985). Whilst these studies have been successful in so far as targeting the kidney is concerned, further measures are required to optimise the delivery of cytotoxic drugs to the tumour rather than normal renal tissue.

Abrams (1964) demonstrated angiographically, in a patient with a hypernephroma, that an arterial infusion of epinephrine redistributes renal arterial blood flow. Ekelund *et al.* (1972) employed similar principles using angiotensin II to increase the diagnostic accuracy of renal angiography. On exposure to vasoactive agents, arterioles supplying normal

**Figure 1** Four posterior images of left kidney in indentical orientation and scale. Top left: posterior view of coronal section of left kidney. Top right: line drawing of coronal section of left kidney; 1: normal superior pole; 2: 'viable' tumour; 3: 'non-viable' tumour; 4: normal inferior pole. Bottom left: post-resection gamma camera image of the distribution of microspheres delivered before angiotensin II. Bottom right: post-resection gamma camera image of the distribution of microspheres delivered after angiotensin II.

tissue constrict whereas tumour blood vessels, which lack smooth muscle, remain dilated. This process therefore diverts renal blood flow and its contents away from normal tissue towards tumour. Lang (1970) used these techniques to deliver therapeutic radioactive pellets to renal tumours. Therefore these techniques allow second level targeting. Our previous pilot experiments, on three renal carcinoma patients, showed that the ratio of radiolabelled microspheres delivered to tumour compared with normal kidney, following an angiotensin II infusion, was 2.5:1, 3:1 and 7.3:1 respectively (unpublished data). Prior to the present study, the improvement in tumour: normal ratio, compared with pre-angiotensin II distribution, was not known. Our results suggest that angiotensin II may improve targeting of renal tumours by 4-fold.

Although radiolabelled microsphere distribution studies reveal changes in blood flow to tumour relative to normal tissue, they do not explain whether these observations are secondary to a decrease in blood flow to normal kidney, increased blood flow to tumour or a combination of both these factors. The introduction of laser-doppler flow equipment has allowed a dynamic study of tissue flow which has revealed an absolute increase in blood flow to tumour following angiotensin II infusion. Unfortunately, we did not possess two probes, therefore we were able to measure simultaneously blood flow to normal kidney and tumour.

In this experiment blood flow was studied using four techniques; *in vivo* perfusion scan, post-resection perfusion scan, tissue well gamma counting and laser-doppler measurements. It is interesting to note that all these methods produced similar results. Therefore *in vivo* renal arterial perfusion scintigraphy reasonably estimates the relative distribution of microspheres to tumour relative to normal kidney. However, the well gamma counts revealed a greater enhancement of tumour: normal ratio than the post-resection scan which, in turn, produced a higher ratio than the *in vivo* scan. These observations may be explained by increased scatter due to the kidney being further from the gamma camera on the *in vivo* scan compared with the post-resection scan. Furthermore, the gamma camera only gives a two dimensional analysis of the kidney (therefore the 'tumour' ROI includes normal tissues anterior and posterior to the tumour) whereas the well counts reflect activity in tumour alone. The well counts, therefore, probably give the most accurate reflection of tumour: normal kidney ratio enhancement. The accuracy of gamma camera imaging may be improved by employing tomographic techniques.

The albumin microspheres in normal kidney were seen to embolise in the afferent arterioles and glomerular capillary loops. The mean biological half-time of these microspheres has been shown to be 2.4 days (Goldberg *et al.*, 1991) and it is therefore likely that these glomeruli would undergo subsequent ischaemic necrosis. Angiotensin II should diminish this problem but it should be born in mind when considering the treatment of patients who have poor renal function or whose contralateral kidney is absent or abnormal.

In conclusion, angiotensin II infusion improves the targeting of regionally administered albumin microspheres to renal tumours. The use of cytotoxic loaded albumin microspheres in the management of renal tumours merits further study.

The authors acknowledge funding received from the Cancer Research Campaign, the Medical Research Council and the Association for International Cancer Research for this project.

### References

ABRAMS, H.L. (1964). Altered drug response to tumour vessels in man. *Nature*, **201**, 167.

EKELUND, L., GOTHLIN, J. & LUNDERQUIST, A. (1972). Diagnostic improvement with angiotensin in renal angiography. *Radiology*, **105**, 33.

GOLDBERG, J.A., WILLMOTT, N.S., ANDERSON, J.H. & 4 others (1991). The biodegradation of albumin microspheres used for regional chemotherapy in patients with colorectal liver metastases. *Nucl. Med. Commun.*, **12**, 57.

HARRIS, D.T. (1983). Hormonal therapy and chemotherapy of renal-cell carcinoma. *Semin. Oncol.*, **10**, 422.

KATO, T., NEMOTO, R., MORI, H., TAKAHASHI, M. & TAMAKAWA, Y. (1981). Transcatheter arterial chemoembolisation of renal cell carcinoma with microencapsulated mitomycin C. *J. Urol.*, **125**, 19.

KERR, D.J., WILLMOTT, N., MCKILLOP, J.H., CUMMINGS, J., LEWI, H.J. & MCARDLE, C.S. (1988). Target organ disposition and plasma pharmacokinetics of doxorubicin incorporated into albumin microspheres after intrarenal arterial administration. *Cancer*, **62**, 878.

LANG, E.K. (1970). Superselective arterial catheterisation of tumours of the urogenital tract: a modality used for perfusion with chemotherapeutic agents and infarction with radioactive pellets. *J. Urol.*, **104**, 16.

LANG, E.K. (1971). Superselective arterial catheterization as a vehicle for delivering radioactive infarct particles to tumours. *Radiology*, **98**, 391.

MCARDLE, C.S., LEWI, H., HANSELL, D., KERR, D.J., MCKILLOP, J. & WILLMOTT, N. (1988). Cytotoxic-loaded albumin microspheres: a novel approach to regional chemotherapy. *Br. J. Surg.*, **75**, 132.

PATEL, N.P. & LAVENGOOD, R.W. (1978). Renal cell carcinoma: natural history and results of treatment. *J. Urol.*, **119**, 722.

WIDDER, K.J., SENYEI, A.E. & RANNEY, D.F. (1979). Magnetically responsive microspheres and other carriers for the biophysical targeting of antitumor agents. *Adv. Pharmacol. Chemother.*, **16**, 213.

WILLMOTT, N., CUMMINGS, J., STUART, J.F.B. & FLORENCE, A.T. (1985). Adriamycin-loaded albumin microspheres: *in vivo* distribution and drug release rate in the rat. *Biopharm. Drug. Dispos.*, **6**, 91.